# The Preparation of Future Statistically Oriented Physicians: A Single-Center Experience in Saudi Arabia

**DOI:** 10.3390/medicina60101694

**Published:** 2024-10-15

**Authors:** Anwar A. Sayed

**Affiliations:** Department of Basic Medical Sciences, Taibah University, Madinah 42353, Saudi Arabia; dsayed@taibahu.edu.sa; Tel.: +966-14-861-8888

**Keywords:** doctors, medical education, PEP, physicians, research, Saudi Arabia, statistics, Taibah University

## Abstract

*Background and Objectives*: Statistics are of paramount significance to physicians as they allow them to critically interpret the medical literature and to contribute to it. However, teaching statistics to medical students and physicians, as well as learning statistics, is nothing short of difficult and anxiety-inducing to a great extent. *Materials and Methods*: In this study, an example of a novel approach to teaching statistics to medical students is introduced at a single college of medicine in Saudi Arabia. In this retrospective report, a new approach that has been developed and delivered to students is described. *Results*: The approach, referred to as the personal experience pathway, is part of a major curriculum change to the MBBS program. The track presents statistics to students as a tool, rather than a subject, that students will need to interpret results, either present in the literature or those of the research projects they are conducting. The outcome of this process has been assessed through measuring students’ scholarly output through student self-reporting and has been followed up over four student cohorts graduating between the years 2019 and 2022. The approach has successfully equipped students with a solid foundation of statistical understanding that has allowed them to publish in peer-reviewed journals. Such scholarly output has increased significantly over the last two years. *Conclusions*: The current study presents a framework through which the detailed curriculum plan could be applied to other medical schools, nationally and internationally, which will better prepare future statistically oriented physicians.

## 1. Introduction

The ever-evolving landscape of medical research necessitates a robust foundation in critical appraisal skills amongst future healthcare professionals. In this rapidly expanding field, staying abreast of the latest advancements and critically evaluating the validity of scientific literature is paramount for delivering optimal patient care. This underscores the imperative for medical students to acquire proficiency in statistics, a cornerstone skillset that empowers them to navigate the complexities of medical research and translate scientific findings into informed clinical decisions [1].

The significance of statistics in medical education is extensively documented in the literature. Studies highlight the ubiquitous role of statistics in modern medical research, emphasizing its integration into both undergraduate and postgraduate medical curriculums. This integration equips students with the necessary tools to appraise research methodologies critically, interpret data, and assess the level of evidence presented [2]. In fact, given the current global approach of evidence-based medicine, it has become apparent that future doctors, i.e., students, should be prepared to tackle scientific literature and be driven to learn statistics in such a manner [3].

Furthermore, research highlights the prevalence of statistics anxiety amongst medical students. This anxiety can impede their willingness to engage with and comprehend statistical concepts [4,5]. Addressing this apprehension through effective pedagogical approaches, such as emphasizing the practical applications of statistics in clinical practice, is crucial to fostering a positive learning experience and ensuring that students grasp the fundamental principles of statistical analysis [6,7].

However, the benefits of incorporating statistics and epidemiology into medical education go far beyond equipping students with the ability to appraise research critically. As highlighted in previous studies, statistical literacy empowers medical students to design and conduct basic research; understanding fundamental statistical concepts allows students to formulate research questions, select appropriate methodologies, and analyze data effectively, contributing to the advancement of medical knowledge [8]. It also enables students to communicate scientific findings. In other words, proficiency in statistics enables students to interpret and explain complex research findings to healthcare professionals and patients clearly and concisely. As the medical field continues to evolve, the ability to critically evaluate new research and integrate findings into clinical practice becomes increasingly important. Furthermore, it also enables doctors to apply statistical concepts in studies that they are conducting themselves. Statistical literacy equips students with the necessary skills to navigate this ever-changing landscape and continuously update their knowledge base.

Despite the numerous benefits, challenges persist when integrating statistics into medical education. Many medical students experience statistics anxiety, stemming from factors such as perceived difficulty, lack of confidence in their mathematical abilities, and negative past experiences with the subject. Additionally, curricular constraints can limit the time dedicated to teaching statistics, potentially hindering students’ ability to grasp the concepts and develop proficiency fully [4,5].

Several solutions have been proposed to overcome these challenges, which have been described in detail and summarized by Leppink [9]. Briefly, these strategies include tailoring statistical education to the needs of medical students: Focusing on the practical applications of statistics in everyday clinical practice can enhance its relevance and address student anxieties. This can be achieved by incorporating real-world examples and case studies that illustrate how statistical concepts are used in various medical scenarios [10]. Also, another strategy is utilizing interactive teaching methods: employing active learning strategies like problem-based learning (PBL) and small group discussions can foster a deeper understanding of statistical concepts and encourage student engagement. Additionally, technology could be integrated into the learning process: utilizing statistical software packages can alleviate some of the computational burden associated with statistics and provide students with valuable hands-on experience in data analysis [9].

This report will present an example of a novel approach of integrating statistics into the Bachelor of Medicine and Surgery (MBBS) program at the College of Medicine at Taibah University (CMTU). The brief report will also highlight how the program managed, through a thorough curricular reform, to equip medical students with the statistical background needed to interpret and contribute to the existing medical literature.

## 2. Materials and Methods

This is a descriptive retrospective study which describes the curricular change in the undergraduate MBBS program from the 2003 curriculum to the 2013 one, with an emphasis on equipping undergraduate medical students with foundational statistical knowledge. The report will continue to describe the impact of such a curricular change, reflected in the number of scholarly outputs by students following the change.

The current undergraduate MBBS program is delivered across 6 years, in which the first 3 years integrate basic medical sciences and clinical teachings. Years 4-6 are entirely of a clinical nature, and the degree is awarded once the students pass their academic years, as well as an obligatory paid one-year clinical internship in which students do 2 month rotations of Medicine, Surgery, Pediatrics, Obstetrics and Gynecology, and Emergency Medicine. Students can also choose 2 months of elective work during the internship year.

During the undergraduate MBBS program, students are supported by a longitudinal mentorship track, known as the Personal and Professional Development track, in which students are assigned a mentor. The mentor assists students in developing an ongoing professional portfolio of personal development and achievements, as well as guiding them on what to expect next in their career. As part of the institutional support provided through the library electronic services, students have access to subscription-based journals, as well as complimentary access to plagiarism detection software. Financially, students are supported with a monthly stipend equivalent to USD 264.

The data described here are the results of 4 undergraduate MBBS student cohorts graduating in the academic years 2018/2019, 2019/2020, 2020/2021, and 2021/2022, representing a total of 617 students, with an almost equal gender distribution. The student cohorts included in this study were the first to be recorded by the MBBS program as part of a newly introduced quality Key Performance Indicator (KPI), which aims to assess student scholarly activity through counting the number of papers published by students. These data were collected through students’ self-reporting to the MBBS program administration, through which students uploaded their unique researcher identifier (ORCID) [11], as well as their published manuscripts’ details. Student scholarly output was calculated by dividing the number of students who had published at least a single article in a peer-reviewed journal during their undergraduate study by the total number of students in the same cohort and then multiplying by 100. This can be better explained through the following formula:

Student scholarly output = (The number of students who have published a single article/ The total number of students in the same cohort) × 100.(1)

Descriptive statistics, including frequency and percentages, have been used to describe categorical data. Data analysis and figure generation were performed using GraphPad Prism Version 10.2.2 for Windows.

Given the nature of this study and the absence of human and animal involvement, and as no personal or sensitive information was recorded in the course of this study, no institutional review board ethical approval was needed for this study.

## 3. Results

### 3.1. The Transformation of the MBBS Curriulum into a Research-Oriented One

The first MBBS curriculum at CMTU was a 6-year program, followed by a clinical internship year in which medical students completed their clinical rotations. The MBBS program introduced at the CMTU had two subjects relevant to statistics and medical research. The first course was Medical Statistics, which was introduced to first-year medical students. The course covered essential topics that included basic descriptive statistics, such as the means, medians, and modes, as well as very few minor analytical statistics, mainly concerned with sensitivity, specificity, and positive and negative predictive values.

The second relevant course that students took as part of their MBBS program was Medical Research Skills. The course was delivered to fourth-year medical students and focused on the different types of studies used in Medicine and their evidential strength in influencing medical decisions. The course also addressed the ethical considerations faced by researchers, including participants’ privacy, confidentiality, right to withdraw, and consenting.

Both of these courses were entirely theoretical and did not include any practical aspects that actively engaged students. Finally, both courses used medical textbooks as references for the students, and students were assessed through multiple-choice question exams.

The new MBBS program introduced a new longitudinal track, i.e., a series of courses that run parallel to the curriculum, called the Personal Experience Pathway (PEP). The PEP track consists of three levels: the first two are taken in the first and second year of the MBBS program, whereas the last level is taken over the last two years. In this parallel track, the students become familiarized with scientific literature and learn how to interact with it and contribute to it. Such a contribution starts from learning academic writing, using reference manager software, and producing academic outputs such as posters, review articles, and original research projects. The details of the PEP curriculum planning are described in Supplementary Table S1.

**Figure 1:**
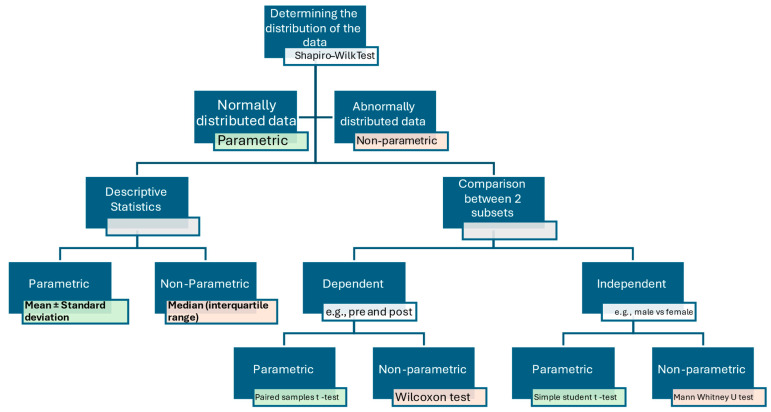
A concept map on how to deal with continuous numerical data. The diagram demonstrates a stepwise approach to tackling continuous numerical data, starting from determining their distribution and the appropriate tests used for each function based on the distribution of the data and the purpose of the statistics used. Green rectangles refer to statistical tools used for parametric data, while orange represents those for non-parametric data.

An essential aspect of the PEP track is the inclusion of statistics as a tool to perform research rather than a separate subject to be studied and examined on, i.e., a means of research rather than an endpoint. In this track, the students are not taught statistics in depth but instead informed about the need to choose the appropriate statistical method and instructed on choosing the proper statistical tests for their research question. For example, students are provided with a concept map for interpreting continuous numerical data, such as patients’ age. This concept map provides students with a systematic approach to handling these data, from determining their distribution (parametric or non-parametric) to, subsequently, how to compare/analyze them based on their research question. An example of this concept map is provided in Figure 1.

### 3.2. The Improvement in Student Scholarly Output Due to the Curricular Change

The data described here are the results of four undergraduate MBBS student cohorts graduating in the academic years 2018/2019, 2019/2020, 2020/2021, and 2021/2022, representing a total of 617 students, all of whom are Saudi Arabians, with an almost equal gender distribution.

**Figure 2:**
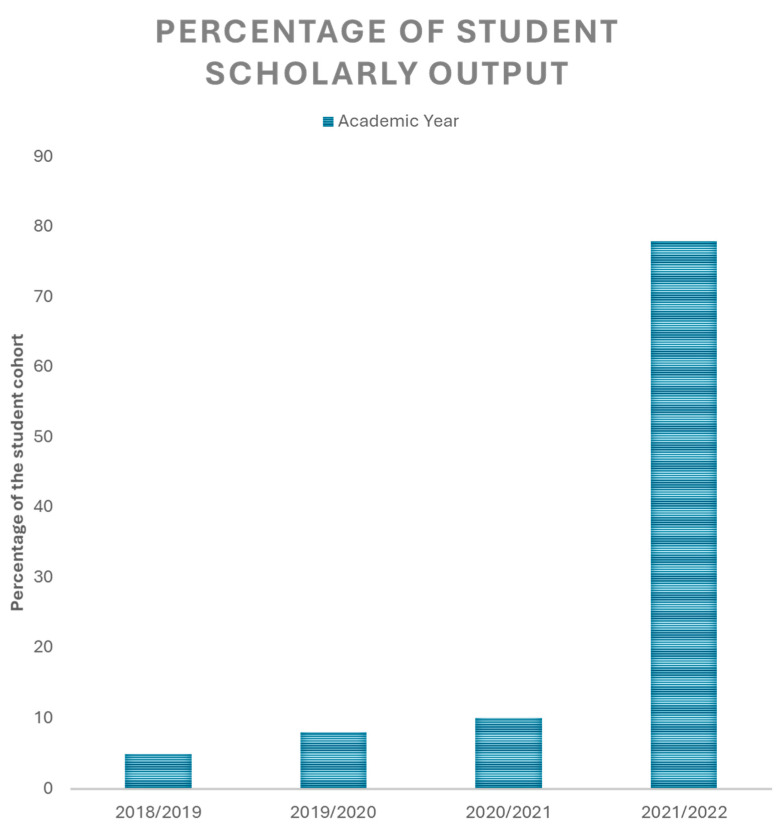
The percentage of the student scholarly output across the different student cohorts. The bar chart demonstrates the percentages of the students who published at least one journal article in a peer-reviewed journal during their undergraduate studies. The bars are across the student cohorts of different academic years between 2018/2019 and 2021/2022.

Following the implementation of the updated PEP track, the program has adopted a new KPI to assess the impact of the PEP track on developing the student’s research and statistical analytical capabilities. This indicator is the student scholarly output, which refers to the percentage of students from their respective cohorts who have published at least a single article in a peer-reviewed journal during their study in the MBBS program. The first measurement recorded under this KPI was in the student cohort graduating in the academic year 2018/2019. Interestingly, a significant increase was observed in the student scholarly output, increasing from less than 10% in the 2018/2019 cohort to almost 80% in the 2021/2022 cohort, as shown in Figure 2.

## 4. Discussion

Statistics is a notoriously troublesome topic for students to teach, learn, and understand. This is more so the case when it is taught to non-specialized students or other disciplines, e.g., medicine or biomedical sciences [12]. Such difficulty raises the need to transform the process of teaching statistics. In this brief report, an example of a college of medicine is presented where the MBBS program had gone through a curricular change that included a significant change in the way in which statistics were taught to the students. And rather than statistics being a separate subject that students take and go through several exams to pass, it is provided to students as a tool needed to conduct scientific research [13].

The presented PEP track provides a tailored curriculum that actively engages students in learning. The participatory nature of the track further enhances students’ learning [14]. Furthermore, the PEP track adopts a gradual approach, which has been suggested previously by Macdougall’s proposed methodology [15]. Moreover, the use of several methods for learning needs assessment ensures triangulation of the results, i.e., ensuring the objectivity of the information obtained from the questionnaire, which is further confirmed and validated through focus groups and a logbook [16,17]. Given their current use and use in previous years, these methods are practical, without difficulties, and can be implemented throughout the program. Another advantage of the presented framework is that it based on Grants’ steps of curriculum planning and design due to its practicality and the ease of following it [18].

Importantly, the PEP track, as part of the MBBS program, delivers key program missions, as well as fits national medical education frameworks. The MBBS program mission “*Promote scientific and clinical research activities*” influences the curriculum aim and the College of Medicine’s mission of “*Fostering scientific research*”. The PEP curriculum’s aim aligns with the Research and Scholarship theme of the Saudi Medical Education Directive’s (SaudiMED) Framework themes [19]. However, similar courses in other MBBS programs across Saudi Arabia share these aims with the PEP track. However, the local research needs of the region of Madinah, where the University is located, are reflected in the PEP curriculum, which differentiates it from these courses [20].

The PEP track has been integrated into the MBBS program. Integration of a curriculum refers to the linkage of different concepts to facilitate students’ learning by providing context and a chance to apply their knowledge [21]. Such integration could be horizontal, i.e., between courses of the same year, or vertical, i.e., across year levels. The revised PEP curriculum is horizontally integrated, i.e., concepts related to scientific literature and its appraisal are also mentioned in the problem-based curriculum, taken concurrently with the PEP track. The PEP curriculum is also vertically integrated as it is delivered across several year levels. Vertical integration is justified by the constructivist theory in which students use their learning as building blocks as they progress through the PEP curriculum [22]. Although it is thought that curriculum integration facilitates students’ learning, it is essential to realize that integration, as Grant suggested, is a mental process completed by students [23]. Staff must consistently remember and highlight such a realization to help students make these connections and apply them in practice. This is also in line with previous studies, which demonstrated that the contextual learning of statistics, such as problem-based or case-based learning [23,24,25] or in a flipped classroom format [26,27] and using advanced software [28], does indeed improve students’ acceptance and subsequently enhance their learning of medical statistics and their satisfaction with such learning [29].

An important premise this study presents is that we do not necessarily need students to have a high level of math skills as a pre-requisite to learn medical statistics. It is, in fact, the approach and the process of handling data that we, as an MBBS program, are interested in. In a similar context, Bodger presented their institutional experience for teaching statistics to undergraduate life science students, in which statistics is taught without a mathematical component [30]. The course was shown to receive very positive feedback and was popular among students.

This study presents an apparent cause of the increased students’ research output, demonstrated by publications. Although this would not be possible without equipping students with the skillset and tools needed to achieve this scholarly level, it is not the sole reason behind the increased interest in publications. The Saudi Commission for Health Specialties (SCFHS) oversees the postgraduate training programs in Saudi Arabia for physicians, dentists, pharmacists, nurses, and laboratory-based specialties. The commission also sets competitive criteria for admission into these programs. Within these criteria, they have recently introduced additional points for participation in research, evidenced by publications in peer-reviewed journals [31]. These points have further increased students’ interest in becoming able to conduct scientific research and publish it in peer-reviewed journals. Nevertheless, this can be taken as a driver for the further increase in student scholarly output but does not negate the impact that the PEP track left on students.

As with any other study, some limitations can be observed in this study. This study presents an experience of a single center, whose circumstances may be different from other institutes. Nevertheless, applying the presented framework and obtaining the expected outcomes in other centers could be possible. Furthermore, single-center studies tend to demonstrate a larger effect compared to multicenter studies [32,33]. It is, therefore, recommended that institutions should carefully analyze the framework’s results and adapt it strategically to their circumstances [34]. Secondly, the outcome that this study is based on, i.e., student scholarly output, is based on students self-reporting and did not differentiate between the different types of studies. For example, a student could have published a narrative review, which barely uses any statistics, and still counted as a positive contribution. Thirdly, the data described in this study are of a retrospective nature and include only four student cohorts. Future studies should address the limitations of this study. They should be carefully designed to be implemented and examined across several medical schools. Additionally, these future studies should carefully analyze the student scholarly output, i.e., take into account the number of original studies, which include the use of statistics, as well as following up on students longitudinally to ensure their firm grasp and application of medical statistics in their future publications.

## 5. Conclusions

The current study presents a pilot evidence-based approach towards preparing future physicians with a solid theoretical statistical background. This background is further reinforced through the students’ use of statistics in their research projects. Such an experience would better prepare physicians to critically interact with scientific literature, as well as actively contribute to it. The presented curriculum mapping provides a detailed yet applicable framework which could be easily adopted by other medical schools in Saudi Arabia. And while the strategy appears effective, it might not be applicable in other educational settings with less support and fewer resources.

## Data Availability

The original contributions presented in the study are included in the article, and further inquiries can be directed to the corresponding author.

## Supplementary Materials

The following supporting information can be downloaded at: https://www.mdpi.com/article/10.3390/medicina60101694/s1, Table S1: The Personal Excellence Pathway (PEP) track curriculum mapping.
