# Supplementary material for: The Preparation of Future Statistically Oriented Physicians: A Single-Center Experience in Saudi Arabia
Source: Medicina (Kaunas). 2024 Oct 15;60(10):1694. doi: 10.3390/medicina60101694 (PMC11509708; doi:10.3390/medicina60101694)
Supplement: Supplementary file 1 [file medicina-60-01694-s001.zip › medicina-3223116-supplementary.pdf]

Table S1. The Personal Excellence Pathway (PEP) track curriculum mapping.

**Learning domain**	**Intended learning outcomes**	**Teaching and learning activities**	**Indicative content**	**Assessments (formative and/or summative)**	**Resources required**
**Knowledge**	Describe plagiarism and how to avoid it.	- Lecture<br>- E-platform<br>- Self-assessment quiz	- Different forms of plagiarism<br>- The University policy on plagiarism<br>- The use of reference manager software, e.g., Mendeley	**Formative**: MCQ quiz on the e-platform<br>**Summative**: Checking the similarity index of the students' reports using iThenticate.	- Institutional iThenticate subscription<br>- Reference manager software
**Cognitive skills**	Critically appraise the medical literature in the selected research domain	- Lectures<br>- Workshops<br>- Student-led tutorial sessions	- Types of studies<br>- CASP checklist<br>- Example papers<br>- E-library	**Formative:** assessment and feedback by both the tutor and peers.<br>**Summative:** Two independent assessors evaluate the final written report (Introduction and discussion).	- Institutional journal subscriptions
	Synthesise and summarise the evidence retrieved in scientific writing	- Group meetings with the supervisor<br>- Workshops<br>- E-platform	- Academic writing workshops	**Formative:** Assessment and feedback by both the tutor and peers.<br>**Summative:**<br>- Supervisor assesses the student's writing.<br>- The final report evaluation (Introduction).	
	- Selecting, applying and discussing appropriate statistical methodology to answer the research question	- Lectures<br>- Workshop	- Descriptive statistics.<br>- Analytical statistics.<br>- Data interpretation<br>- Data presentation	**Formative:** Midcourse presentation.<br>**Summative:**<br>- Supervisor assesses the student's writing.<br>- The final report evaluation (Methods and Results).	- Institutional subscription to statistical software<br>- Campbell, M. D., & Swinscow, T. D. V. (2009). Statistics at Square One (11th ed.).

	Interpret and critique the evidence retrieved from own work in the context of existing knowledge.	- Group meetings with the supervisor<br>- Workshops<br>- E-platform	Academic writing	**Formative**:<br>- Supervisor and peer feedback<br>**Summative**:<br>- Supervisor assesses the student's writing.<br>- The final report evaluation (Discussion).	
**Interpersonal skills and Responsibility**	Demonstrate reasoning skills through the ability to define problems and use available information to detect gaps and limitations of medical knowledge.	- Group meetings with the supervisor<br>- Workshops<br>- E-platform	Academic writing	**Formative**:<br>Supervisor qualitative feedback report<br>**Summative**:<br>- The final report evaluation (Introduction).	Institutional subscription of statistical software
	Reflection on the ethical dilemmas and fieldwork dilemmas during the research process conduction	- Lectures<br>- E-platform	Institutional IRB policies.	**Formative**:<br>Supervisor feedback report<br>**Summative**:<br>The obtainment of an IRB ethical approval is a requirement.	IRB Research Ethics Committee
	Apply time management strategies throughout the phases of the research project	- Lectures<br>- Workshop<br>- E-platform<br>- Frequent communications (reminders)	-Gantt Chart<br>- Time management skills	**Formative**:<br>Supervisor feedback report<br>**Summative**:<br>- Zero policy for late submissions (late submissions will not be marked).<br>- Timed oral presentation is limited. 20% of the mark is dedicated to group adherence to the time limit.	
**Communication and Numerical skills**	- Search the electronic literature systematically<br>- Recognise the best evidence available to answer a question	- Lectures<br>- Workshops	- Strength of the evidence in the medical literature<br>- Using the E-library	**Formative**:<br>Feedback from supervisors and peers<br>**Summative**:<br>- The final report evaluation (Study	Institutional journal subscriptions

			- The university's digital resources	design and methodology).	
	Communicate effectively with supervisor and peers in a scientific project	- Workshops	- Communication skills	**Formative**: Feedback from supervisors and peers	Institutional email
	Use of required technology-based research tools in carrying out data collection, analysis and management	- Workshops<br>- E-platform	- Institutional data management policy<br>- Ethical concerns on the use and storage of research data.	**Formative**: Feedback from supervisors and peers<br>**Summative**:<br>- The final report evaluation (Data management and statistical analysis).	-Institutional subscription to cloud storage.<br>- Statistics software<br>- On-campus secure computers

MCQ: Multiple choice questions.
